# Impaired Vascular Contractility and Aortic Wall Degeneration in Fibulin-4 Deficient Mice: Effect of Angiotensin II Type 1 (AT_1_) Receptor Blockade

**DOI:** 10.1371/journal.pone.0023411

**Published:** 2011-08-09

**Authors:** Els Moltzer, Luuk te Riet, Sigrid M. A. Swagemakers, Paula M. van Heijningen, Marcel Vermeij, Richard van Veghel, Angelique M. Bouhuizen, Joep H. M. van Esch, Stephanie Lankhorst, Natasja W. M. Ramnath, Monique C. de Waard, Dirk J. Duncker, Peter J. van der Spek, Ellen V. Rouwet, A. H. Jan Danser, Jeroen Essers

**Affiliations:** 1 Division of Vascular Medicine and Pharmacology, Department of Internal Medicine, Erasmus MC, Rotterdam, The Netherlands; 2 Department of Cardiology, Thoraxcenter, Erasmus MC, Rotterdam, The Netherlands; 3 Department of Cell Biology and Genetics, Cancer Genomics Center, Erasmus MC, Rotterdam, The Netherlands; 4 Department of Bioinformatics, Erasmus MC, Rotterdam, The Netherlands; 5 Department of Pathology, Erasmus MC, Rotterdam, The Netherlands; 6 Department of Vascular Surgery, Erasmus MC, Rotterdam, The Netherlands; 7 Division of Experimental Cardiology, Department of Cardiology, Thoraxcenter, Erasmus MC, Rotterdam, The Netherlands; 8 Department of Radiation Oncology, Erasmus MC, Rotterdam, The Netherlands; Leiden University Medical Center, Netherlands

## Abstract

Medial degeneration is a key feature of aneurysm disease and aortic dissection. In a murine aneurysm model we investigated the structural and functional characteristics of aortic wall degeneration in adult fibulin-4 deficient mice and the potential therapeutic role of the angiotensin (Ang) II type 1 (AT_1_) receptor antagonist losartan in preventing aortic media degeneration. Adult mice with 2-fold (heterozygous Fibulin-4^+/R^) and 4-fold (homozygous Fibulin-4^R/R^) reduced expression of fibulin-4 displayed the histological features of cystic media degeneration as found in patients with aneurysm or dissection, including elastin fiber fragmentation, loss of smooth muscle cells, and deposition of ground substance in the extracellular matrix of the aortic media. The aortic contractile capacity, determined by isometric force measurements, was diminished, and was associated with dysregulation of contractile genes as shown by aortic transcriptome analysis. These structural and functional alterations were accompanied by upregulation of TGF-β signaling in aortas from fibulin-4 deficient mice, as identified by genome-scaled network analysis as well as by immunohistochemical staining for phosphorylated Smad2, an intracellular mediator of TGF-β. Tissue levels of Ang II, a regulator of TGF-β signaling, were increased. Prenatal treatment with the AT_1_ receptor antagonist losartan, which blunts TGF-β signaling, prevented elastic fiber fragmentation in the aortic media of newborn Fibulin-4^R/R^ mice. Postnatal losartan treatment reduced haemodynamic stress and improved lifespan of homozygous knockdown fibulin-4 animals, but did not affect aortic vessel wall structure. In conclusion, the AT_1_ receptor blocker losartan can prevent aortic media degeneration in a non-Marfan syndrome aneurysm mouse model. In established aortic aneurysms, losartan does not affect aortic architecture, but does improve survival. These findings may extend the potential therapeutic application of inhibitors of the renin-angiotensin system to the preventive treatment of aneurysm disease.

## Introduction

Degeneration of the medial layer of the aorta is a key feature of aneurysm disease and aortic dissection [1]. Cystic medial degeneration is characterized by elastic fiber fragmentation, loss of smooth muscle cells (SMC), and accumulation of amorphous extracellular matrix (ECM) in the aortic wall. Although media degeneration occurs to some degree with aging, excessive aortic wall degeneration may lead to dilatation of the aorta and aneurysm formation, or, alternatively, aortic dissection [2], [3]. In addition, advanced aortic degeneration may be part of inherited disorders of the connective tissue. One of the most common of these syndromes is Marfan syndrome (MFS), resulting from a mutation in the FBN1 gene which encodes the ECM glycoprotein fibrillin-1 [4]. MFS is characterized by elastic fiber fragmentation, loss of elastin content, and accumulation of amorphous matrix components in the aortic wall, resulting in the formation of thoracic aortic aneurysms (TAAs) [5]. Mice with a mutation in the fibrillin-1 gene are widely used to study the pathophysiologic mechanisms underlying MFS and its treatment options [6].

Several mutations in other genes encoding extracellular matrix proteins have also been identified in patients with TAAs, including mutations in the fibulin-4 gene [7] [8]. Fibulin-4 is one of the seven-member family of ECM proteins that play a role in elastic fiber assembly and function [9]. Fibulin-4 is highly expressed in the medial layers of blood vessel walls, including the aortic media [10]. It has been shown that mutant mice lacking fibulin-4 (Fibulin-4^-/-^) die perinatally from aortic rupture [11]. Furthermore, newborn mice with a systemic 4-fold reduced expression of fibulin-4 (Fibulin-4^R/R^) display elastic fiber fragmentation and develop aneurysms in the ascending thoracic aorta. Interestingly, even a 2-fold reduced expression of fibulin-4 in the heterozygous Fibulin-4^+/R^ mice already induces similar, though milder, changes in the aorta [12].

Since aneurysm disease is a condition of the aging population, the present study first focused on the structural and functional characterization of aortic wall degeneration in adult fibulin-4 deficient mice. Recent studies have shown that antagonizing transforming growth factor-β (TGF-β) by either TGF-β neutralizing antibodies or the angiotensin (Ang) II type 1 (AT_1_) receptor antagonist losartan can slow the progression rate of aortic root dilatation in an MFS mouse model [6] and in patients with MFS [13]. Therefore, we next investigated the role of the renin-angiotensin system (RAS) in aneurysm formation in fibulin-4 deficient mice. We show that prenatal treatment with the AT_1_ receptor blocker losartan can prevent aortic media degeneration in this non-MFS aneurysm mouse model. Losartan could not attenuate established aortic aneurysms in adult fibulin-4 mice, but largely improved survival of these animals. These findings point towards potential therapeutic application of inhibitors of the RAS to the preventive treatment of aneurysm disease.

## Methods

### Experimental animals

We previously generated a fibulin-4 allele with reduced expression by transcriptional interference through placement of a TKneo targeting construct in the downstream Mus81 gene [12]. Heterozygous (Fibulin-4^+/R^) mice in a mixed C57Bl/gJ;129Sv background were mated to obtain Fibulin-4^+/+^, Fibulin-4^+/R^ and Fibullin-4^R/R^ littermates and were housed in the institutional animal facility. All experiments were performed under the regulation and permission of the Animal Care Committee of the Erasmus MC, Rotterdam, The Netherlands (protocol ID 139-08-06). The investigation conforms to the *Guide for the Care and Use of Laboratory Animals* published by the US National Institutes of Health (NIH Publication No. 85-23, revised 1996).

### Histology and immunohistochemistry

Mice (age 100 days) were euthanized by an overdose CO_2_, fixed by perfusion fixation with 4% formaldehyde, and autopsied according to standard protocols. Perfusion-fixed aortas were isolated and paraffin embedded. Next, 4 µm sections were haematoxylin and eosin stained and stained for elastin (Verhoeff-van Gieson), glycosaminoglycans (Alcian Blue) and SMCs (α-SMA). Immunohistochemistry for phosphorylated Smad2 (pSmad2) was performed as described previously [14] using rabbit antiphospho-smad2 antibodies. The relative SMCs area of the ascending aorta was quantified by calculating the surface area of SMCs divided by the total surface area of the aortic rings (Qwin, Leica, Gleisburg, Switzerland). The relative amount of positive stained pSmad2 cells was calculated as the amount of positive stained pSmad2 cells, divided by the total number of cells.

### Hemodynamic measurements

Mice (15–20 weeks old) were sedated with 4% isoflurane and intubated as previously described [15]. For measuring systolic and diastolic BP, mice were instrumented with a calibrated high fidelity 1.4 Fr microtip pressure transducer catheter (SPR-671, Millar Instruments), which was inserted into the left carotid artery and advanced into the aortic arch [12]. Hemodynamic data were recorded and digitized using an online 4-channel data acquisition program (ATCODAS, Dataq Instruments, Akron, Ohio, USA), for later analysis with a program written in Matlab. Ten consecutive beats were selected for determination of BP.

### Mulvany myographs

Male mice (age 120 days) were euthanized with an overdose of pentobarbital i.p. (60 mg/kg). Thoracic aorta, abdominal aorta and iliac artery were isolated and stored overnight in cold, oxygenated Krebs-Henseleit buffer solution. The following day, vessel segments were mounted in 6-mL organ baths (Danish Myograph Technology, Aarhus, Denmark) containing Krebs-Henseleit buffer (NaCl 118, KCl 4.7, CaCl_2_ 2.5, MgSO_4_ 1.2, KH_2_PO_4_ 1.2, NaHCO_3_ 25 and glucose 8.3; pH 7.4) at 37°C and oxygenated with 95% O_2_ and 5% CO_2_. The tension was normalized to 90% of the estimated diameter at 100-mm Hg effective transmural pressure.[16] Maximum contractile responses were determined using 100 mmol/L KCl. Concentration response curves (CRCs) were constructed to phenylephrine and Ang II (Sigma); the latter with a 30-minute incubation with the NO synthase inhibitor L-NAME (100 µmol/L; Sigma).

### Microarray hybridizations

Standard procedures were used to obtain total RNA (Qiagen) of two Fibulin-4^+/+^, two Fibulin-4^+/R^ and four Fibulin-4^R/R^ aortas (10 days old). Synthesis and hybridization was performed as described before [12]. To examine the quality of the various arrays, several R packages (including affyQCreport) were run starting from the CEL files. All created plots, including the percentage of present calls, noise, background, and ratio of GAPDH 3′ to 5′ (<1.4) indicated a high quality of all samples and an overall comparability, except for two samples, which were excluded from further analysis. Of the 45101 probe sets, ∼55% was called present in all samples. Raw intensities values of all samples were normalized by robust multichip analysis normalization (background correction and quantile normalization) using Partek version 6.4 (Partek Inc., St. Louis, MO). The normalized data file was transposed and imported into OmniViz version 6.0.1 (Biowisdom, Ltd., Cambridge, UK) for further analysis. For each probe set, the geometric mean of the hybridization intensities of all samples was calculated. The level of expression of each probe set was determined relative to this geometric mean and ^2^log transformed. The geometric mean of the hybridization signal of all samples was used to ascribe equal weight to gene expression levels with similar relative distances to the geometric mean. Differentially expressed genes were identified using ANOVA (Partek) and SAM (OmniViz). Cut-offs values for significantly expressed genes were the FDR and a fold change of 1.5. Functional analysis was done using IPA (Ingenuity, Mountain View, CA). Microarray experiments have been previously described and complied with the regulations for Minimum Information of Microarray Experiments (MIAME) and can be retrieved from ArrayExpress (www.ebi.ac.uk/arrayexpress/ , accession code: E-MEXP-840) [12].

### Biochemical measurements

Kidneys were excised and blood was collected from the left ventricle and stored in 4 mol/l guanine thiocyanate as described before [17]. Both were immediately frozen in liquid nitrogen and stored at −80°C. Ang II was determined using radioimmunoassay, following SepPak extraction and high-performance liquid chromatography separation [18].

### Quantitative real-time reverse transcription polymerase chain reaction

Total RNA was isolated from kidneys and aortic arches using RNeasy Fibrous Tissue Mini Kit (Qiagen) and reverse transcribed using the SuperScript VILO cDNA synthesis kit (Invitrogen). The resulting cDNA was amplified in 40 cycles (denaturation at 95°C for 10 min; thermal cycling at 95°C for 15 sec, annealing/extension at 60°C for 1 min) with a Step-One cycler using TaqMan Universal Mastermix and TaqMan probes (Applied Biosystems) of individual genes. Specific primers (Rplp0 Mm01974474_gH, Efemp2 Mm00445429_m1, Agtr1a Mm00616371_m1, Agtr1b Mm02620758_s1 and Agtr2 Mm01341373_m1) were obtained from Applied Biosystems. After PCR cycling, the fluorescence intensities of the reporter (FAM) dyes were quantified. The threshold cycle (Ct), i.e. the cycle number at which the amount of the amplified gene of interest reached a fixed threshold, was determined subsequently. The comparative Ct method (ΔΔCT) was used for relative quantification of gene expression [19].

### Treatment

Fibulin-4^+/R^ mice were bred to produce Fibulin-4^+/+^ and Fibulin-4^R/R^ mice. Pregnant mice received either propranolol (0.5 g/liter, Sigma), losartan (0.6 gram/liter, Sigma) or placebo in their drinking water as described before [6]. Treatment was started at embryonic day (E)14.5 and continued for five days. At E19.5 the pregnant mice were euthanized by an overdose CO_2_ and a caesarian section was performed to collect the fetuses. Adult Fibulin-4^R/R^ mice and their wild type littermates were treated during 10–14.5 weeks, starting at the age of 5.5 weeks. Aortas from the fetuses and adult mice were isolated and paraffin embedded. Next, 4-µm sections were stained for elastin (Verhoeff-van Gieson). Ascending aortic wall thickness is the average of four measurements per quartile using Leica QWin software (Leica, Glattburg, Switzerland).

### Data-analysis

Normally distributed data are presented as mean±SEM. CRCs were analyzed using Graph Pad Prism 5 (Graph Pad Software Inc., San Diego, California, USA) to determine the maximum effect (*E*_max_) as described before.[20] Analysis of the differences between CRCs was performed by two-way ANOVA. The one-way ANOVA was considered for the analysis of *E*_max_, blood pressures, angiotensin II levels and vessel wall thickness. Both analyses were followed by post hoc evaluation according to Bonferroni. To compare the observed distributions of the genotypes with the expected mendelian distribution, a chi-square test was used. The survival of fibulin-4 mice over time is presented in a Kaplan-Meier curve for a cohort of mice alive at age 3 weeks and the curves were compared by the Log Rank test. To evaluate the dose-dependent effect of fibulin-4 expression, a linear regression analysis was performed to obtain a p for trend. The latter statistical analyses were performed using SPSS 15.0 for Windows (SPSS, Chicago, Ill, USA). All statistical tests were two-sided and a p-value <0.05 was considered statistically significant.

## Results

### Adult fibulin-4 deficient mice display aortic wall degeneration

**Figure 1:**
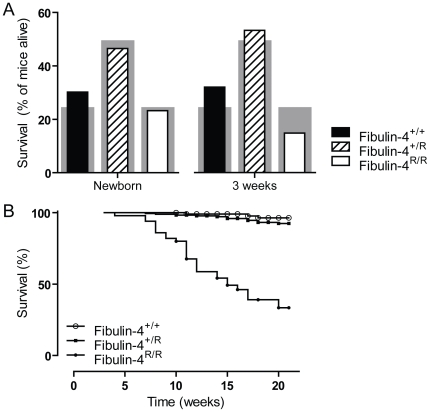
Survival of fibulin-4 mice. (A) Distribution of the three genotypes at 0 and 3 weeks of age. The grey bars show the expected Mendelian distribution and the overlying bars the observed distribution of the different genotypes. Fibulin-4 mice are born in a Mendelian distribution (n = 10–20). Already after three weeks, this distribution is lost (n = 50–180, p<0.0001). (B) Kaplan-Meier survival curves of Fibulin-4^+/+^, Fibulin-4^+/R^ and Fibulin-4^R/R^ mice alive at the age of three weeks (n = 50–180). After 21 weeks, 96% of wild type Fibulin-4^+/+^ and 92% of Fibulin-4^+/R^ mice survived. Survival of Fibulin-4^R/R^ mice dramatically decreased to 33% (p<0.0001 vs. wild type). Note that the survival curve starts with all mice alive at the age of three weeks. Symbols indicate censored data.

**Figure 2:**
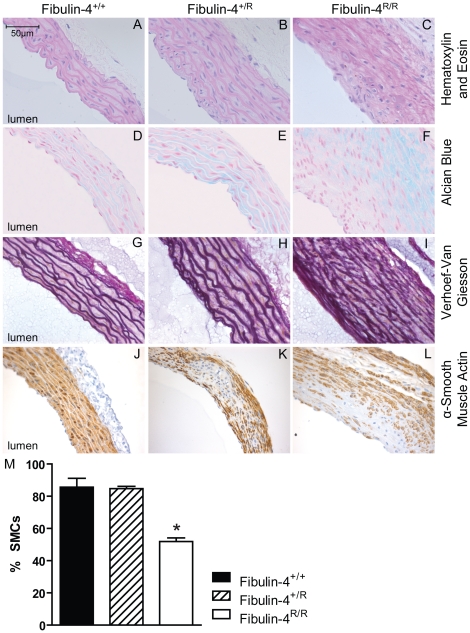
Architecture of ascending thoracic aortas. In adult Fibulin-4^+/R^ and Fibulin-4^R/R^ aortas there is an increase in aortic wall thickness (A–C), glycosaminoglycan depositions (blue areas) (D–F), elastic fiber fragmentation (G–I) and loss of smooth muscle cells in the media (J–L), also quantified (M).

Newborn Fibulin-4^R/R^ mice already showed severe TAAs [12], but only a small number of Fibulin-4^R/R^ mice survived towards adult age. Fig. 1 shows the survival of fibulin-4 deficient mice. Newborn fibulin-4 mice demonstrate a Mendelian distribution of the three genotypes (30.2% Fibulin-4^+/+^, 46.5% Fibulin-4^+/R^ and 23.3% Fibulin-4^R/R^ mice). Due to the high mortality in the first weeks, genotyping takes place at the age of three weeks. At this age, the amount of Fibulin-4^R/R^ mice already dropped to 15% and the Mendelian distribution is lost (Fig. 1A, p<0.0001). To get insight in the mortality rate of these mice, we constructed a Kaplan-Meier curve with all mice alive at the age of three weeks (Fig. 1B). The curves clearly demonstrate a dramatic survival of Fibulin-4^R/R^ mice when compared to their wild type littermates (p<0.0001). The structural alterations resulting from reduced fibulin-4 in adult mice were characterized in 100-days-old mice. All aneurysms of Fibulin-4^R/R^ mice were located in the ascending thoracic aorta. Aortic wall thickness was increased in Fibulin-4^+/R^ and Fibulin-4^R/R^ as compared with Fibulin-4^+/+^ mice (Fig. 2A–C). The increase in aortic wall thickness was, at least in part, due to increased deposition of glycosaminoglycans in the ECM, as demonstrated by Alcian blue staining (Fig. 2D–F). Aortas of wild type mice displayed a normal pattern of elastic lamellae forming dense parallel sheets. In contrast, the thickened aortic walls in fibulin-4 deficient mice displayed changes in elastic fiber organization, varying from moderate elastic fiber fragmentation in Fibulin-4^+/R^ mice to complete destruction of elastin lamellar organization in Fibulin-4^R/R^ mice (Fig. 2G–I). In addition to changes in elastin structure, aortic walls of Fibulin-4^+/R^ and Fibulin-4^R/R^ mice displayed loss of SMCs, as evidenced by α-smooth muscle actin (SMA) staining (Fig. 2J–L) and increased numbers of apoptotic cells (data not shown). Next, to evaluate the reduction of SMCs seen in Fibulin-4^R/R^ aortas, we quantified the amount of SMCs relative to the vessel wall area. Although the absolute amount of SMCs varied among the different genotypes, the relative amount of SMCs was significantly lower in Fibulin-4^R/R^ mice when compared to Fibulin-4^+/+^ and Fibulin-4^+/R^ mice (Fig. 2M).

### Functional consequences of fibulin-4 deficiency

#### Increased aortic pulse pressure

**Figure 3:**
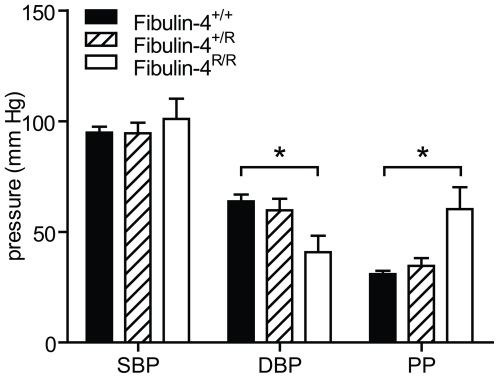
Systolic blood pressure (SBP) and diastolic blood pressure (DBP) measured using an intra-aortic microtip pressure transducer catheter. With decreasing expression of Fibulin-4, DBP decreased and pulse pressure (PP) increased (p for trend 0.009 and <0.001 resp.). Data are mean±SEM of 4–17 mice. *p<0.05 vs. Fibulin-4^+/+^ and Fibulin-4^+/R^ (two-way ANOVA).

Since elastic fiber fragmentation may be associated with loss of elasticity and increased stiffness of the aortic wall, we next determined the *in vivo* aortic blood pressure using a microtip pressure catheter. In Fibulin-4^R/R^ mice a slightly increased systolic blood pressure and decreased diastolic blood pressure was observed compared to wild type animals resulting in a significantly higher aortic pulse pressure in Fibulin-4^R/R^ mice compared to controls (Fig. 3), which is consistent with increased arterial stiffness [21]. Interestingly, we observed a gene dose-dependent decrease (trend) in diastolic blood pressure and increase (trend) in pulse pressure (Fig. 3), while no aortic valve abnormalities are present in Fibulin-4^+/R^ mice. We therefore hypothesize that this blood pressure effect is due to primary vessel wall impairment in fibulin-4 deficient mice, while in Fibulin-4^R/R^ mice, this phenotype is aggravated due to aortic valve dysfunction.

#### Reduced aortic contractility

To evaluate the functional effects of SMC loss, *in vitro* vascular contractility was studied in different segments of the aorta and the iliac arteries. After mounting, the vessel diameter was measured for each segment. Ascending thoracic aortic diameters were 1106±22, 1086±27 and 2023±88 µm for Fibulin-4^+/+^, Fibulin-4^+/R^ and Fibulin-4^R/R^ mice respectively (n = 14–18). Descending thoracic aortic diameters were 830±17, 797±17 and 955±49 µm for Fibulin-4^+/+^, Fibulin-4^+/R^ and Fibulin-4^R/R^ mice respectively (n = 17–22). Abdominal aortic diameters were 568±12, 585±15 and 611±30 µm for Fibulin-4^+/+^, Fibulin-4^+/R^ and Fibulin-4^R/R^ mice respectively (n = 18–20). Iliac arteries were 420±12, 401±11 and 378±14 µm for Fibulin-4^+/+^, Fibulin-4^+/R^ and Fibulin-4^R/R^ mice respectively (n = 18–19). The diameter of both the ascending and descending thoracic aorta were significantly larger in Fibulin-4^R/R^ mice when compared to wild type Fibulin-4^+/+^ mice, while the iliac arteries were significantly smaller in diameter. Furthermore, the increase in vessel diameter of the ascending thoracic aorta was accompanied by an approximately 2-fold elongation of the aortic segment.

**Figure 4:**
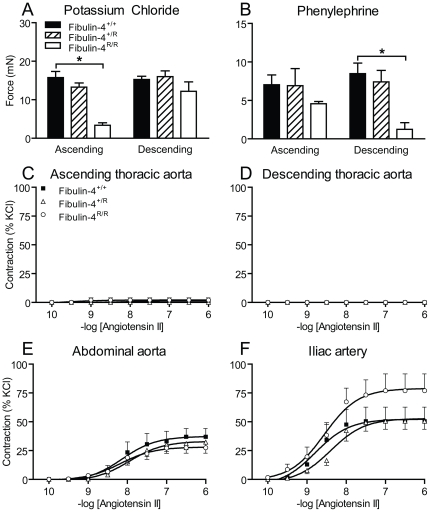
Contractility mediated by KCl, phenylephrine and angiotensin II. (A) In ascending aortas, KCl-induced contractility decreased in a gene dose-dependently in Fibulin-4^+/R^ and Fibulin-4^R/R^ mice (p for trend <0.001). (B) In descending aortas, phenylephrine-induced contractility decreased gene dose-dependently in Fibulin-4^+/R^ and Fibulin-4^R/R^ mice (p for trend 0.004). Data are mean±SEM of 6–18 experiments, *p<0.05 vs. Fibulin-4^+/+^ mice. (C–F) Effect of angiotensin II on (C) ascending thoracic aortas, (D) descending thoracic aortas, (E) abdominal aortas and (F) iliac arteries. Data (mean±SEM of 3–6 experiments) are shown as a percentage of the response to 100 mmol/L KCl.

In line with the relative reduction of SMCs in the thoracic aorta, the maximum contractility of thoracic aortas in response to KCl (100 mmol/L) was more than 3-fold lower in Fibulin-4^R/R^ mice than in Fibulin-4^+/+^ mice (Fig. 4A). Similarly, receptor-mediated vasoconstriction in response to phenylephrine (100 µmol/L) was significantly lower in thoracic aortic rings of Fibulin-4^R/R^ mice than in Fibulin-4^+/+^ mice (Fig. 4B). The contractile responses of the abdominal aorta and the iliac arteries did not differ between groups (data not shown). Increasing doses of Ang II, following a 30-minute incubation with *N* ^ω^-nitro-L-arginine methyl ester (L-NAME), did not induce vasoconstriction in the thoracic aorta (Fig. 3C–D). The contractile responses of the abdominal aorta and iliac arteries in response to Ang II were not different between fibulin-4 deficient and wild type mice (Fig. 4E–F). This difference probably relates to the lower AT_1_ receptor levels in the thoracic aorta than in other large arteries in the mouse [22], [23].

### Disturbed calcium signaling in fibulin-4 deficient mice

**Figure 5:**
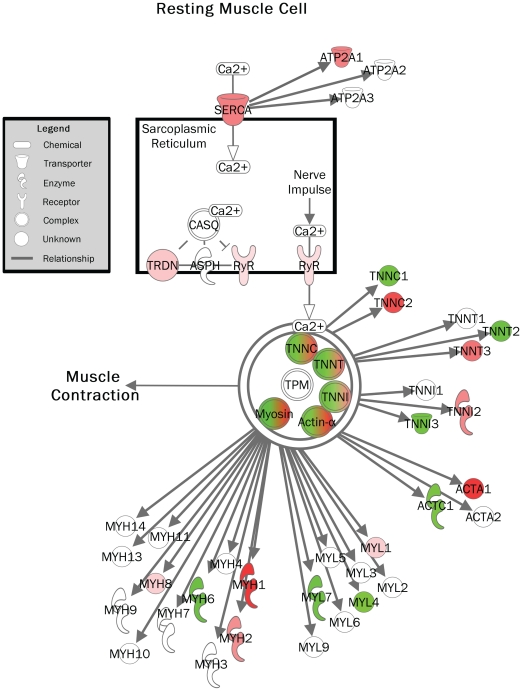
Calcium signaling pathway in a resting muscle cell (Fibulin-4^+/R^ vs. Fibulin-4^+/+^ aortas). Colors show up- (red) and downregulation (green) of molecules involved in muscle cell contraction.

Next, genome-scaled network analysis from Fibulin-4+/+, Fibulin-4+/R and Fibulin-4R/R aortas was performed using dedicated microarray statistics with a focus on canonical pathway analysis. Differentially expressed genes were initially identified using statistical analysis of microarrays ANOVA (false discovery rate (FDR) 0.5 and 1.5-fold change up- or downregulation). Transcriptomes of Fibulin-4+/+ and Fibulin-4+/R full length aortas were compared and 26 probe sets were identified. With Ingenuity Pathway Analysis (IPA), a list of involved canonical pathways was constructed (Supplemental Table S1). The calcium signaling showed up as the top canonical pathway. Next, an independent SAM analysis was performed (FDR of 0.0032 (falsely called <1) and 1.5-fold change up- or downregulation). This approach identified 279 probe sets, from which a second top list of canonical pathways was constructed (Supplemental Table S2). Again, the calcium signaling pathway was highly significant. Interestingly, very specific genes involved in muscle cell contraction were up- or downregulated (Fig. 5).

Next, differences between transcriptomes of Fibulin-4^+/+^ and Fibulin-4^R/R^ aortas were analyzed. Statistical analysis of microarrays ANOVA was performed with the same selection criteria as for Fibulin-4^+/+^ vs. Fibulin-4^+/R^ aortas. Canonical pathway analysis identified mainly pathways involved in immunological and inflammatory diseases (Supplemental Table S3) and after analysis with SAM (FDR 0.2 and 1.5-fold change up- or downregulation) a table with principally similar pathways was constructed (Supplemental Table S4). These analyses identified a few genes involved in the aforementioned calcium signaling pathway.

### Fibulin-4 deficient mice show dysregulation of TGF-β signaling and increased tissue angiotensin II

**Figure 6:**
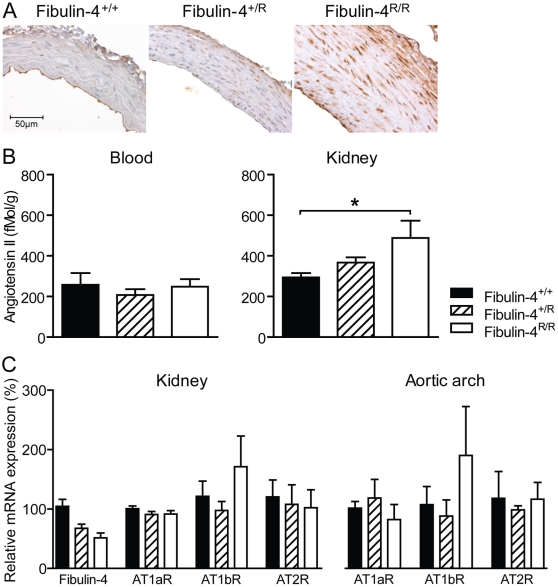
Increased levels of pSmad2 and angiotensin II in fibulin-4 mutant aortas. (A) Immunhistochemistry reveals a graded increase in expression and nuclear translocation of pSmad2 in the aortic media of adult fibulin-4 deficient mice. (B) With reduced fibulin-4 expression, tissue (but not blood) Ang II levels increase (*p* for trend 0.004). Data are shown as mean±SEM of 4–18 experiments. *p<0.05 vs. Fibulin-4^+/+^. (C) Relative mRNA expression of Fibulin-4 and Ang II receptors. As published previously, a substantial decrease of fibulin-4 was observed in Fibulin-4^+/R^ and Fibulin-4^R/R^ mice when compared to wild type littermates. Both the renal and aortic arch AT_1_b receptor content was larger in Fibulin-4^R/R^ mice when compared to Fibulin-4^+/+^ and Fibulin-4^+/R^ mice. No differences in AT_1_a or AT_2_ receptor expression were observed between the different genotypes (n = 3–10). AT1aR, angiotensin II type 1a receptor; AT1bR, angiotensin II type 1b receptor; AT2R, angiotensin II type 2 receptor.

In a mouse model of MFS, it has been demonstrated that dysregulation of TGF-β activation and the RAS play an important role in aneurysm formation [6], [24], [25]. Hence, we next investigated the involvement of TGF-β signaling and Ang II in fibulin-4 deficient mice. First, genome-scaled network analysis from Fibulin-4^+/+^, Fibulin-4^+/R^ and Fibulin-4^R/R^ aortas identified the upregulation of TGF-β in Fibulin-4^R/R^ mice compared to Fibulin-4^+/+^ mice (Supplemental Table S3 and S4). Next, immunohistochemical staining for phosphorylated Smad2 (pSmad2), an intracellular mediator of the TGF-β signal, in ascending thoracic aortas was performed. A graded increase in the nuclear translocation of pSmad2 in the aortic media of Fibulin-4^+/R^ and Fibulin-4^R/R^ mice was observed (Fig. 6A), indicating increased TGF-β signaling in adult aneurysmal fibulin-4 deficient mice.

Ang II is important in TGF-β signaling, by stimulating TGF-β1 mRNA and protein expression, which leads to TGF-β activation. This indicates that TGF-β acts downstream of Ang II signaling [26]. Therefore, we subsequently measured Ang II levels in blood and in kidney tissue of fibulin-4 deficient mice. Plasma Ang II levels were identical in the three genotypes (Fig. 6B). In contrast, renal tissue Ang II levels displayed a clear gene dose-dependent increase in Fibulin-4^+/R^ and Fibulin-4^R/R^ mice (Fig. 5b; p<0.004 for gene deletion effect), which may be due to increased AT_1_ receptor binding at this site, resulting in increased receptor-bounded Ang II levels [27]. Subsequent analysis of angiotensin receptor expression indeed demonstrated increased AT_1_b receptor expression in both the kidneys and aortic arches (Fig. 6C). It is thus reasonable to assume that the Ang II content is also larger in the vasculature of fibulin-4 deficient mice, due to an increased receptor density at this site.

### Treatment with AT_1_ receptor blocker losartan prevents aortic wall degeneration, but does not attenuate established aortic aneurysms

**Figure 7:**
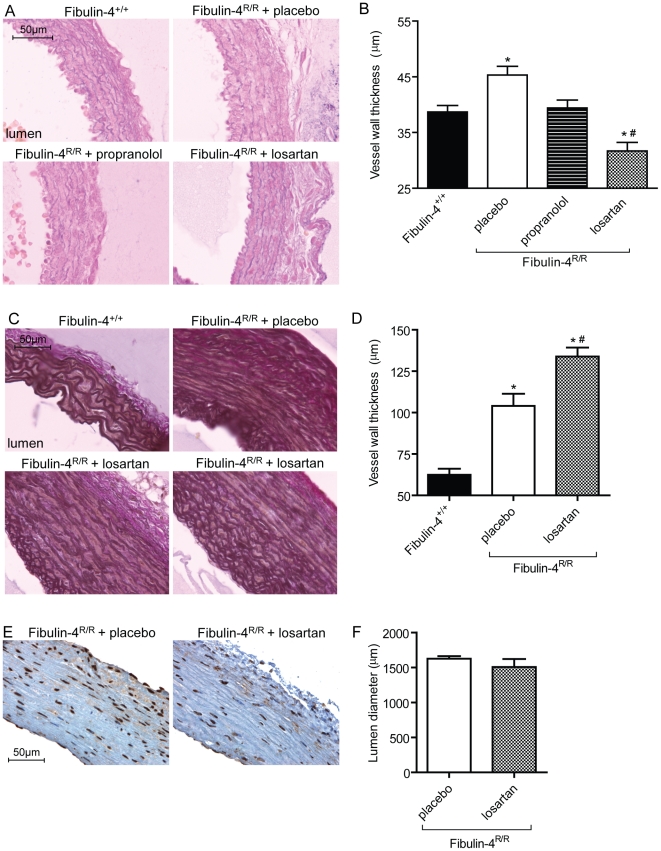
Aortic aneurysm treatment with losartan. (A) Elastic fiber fragmentation in newborn Fibulin-4^R/R^ mice could be prevented with losartan, but not with propranolol or placebo. (B) Vessel wall thickness of thoracic aortas from newborn Fibulin-4^+/+^ and treated Fibulin-4^R/R^ mice. Losartan treatment of Fibulin-4^R/R^ mice recovered vessel wall thickness. (C) Losartan treatment of adult Fibulin-4^R/R^ mice did not improve elastic fiber fragmentation. (D) Vessel wall thickness increased after postnatal losartan treatment. (E) Postnatal treatment with losartan did not reduce the amount of pSmad2 positive cells, nor did it affect lumen diameter (F). *p<0.05 vs. wild type, #p<0.05 vs. placebo-treated Fibulin-4^R/R^ mice, n = 4–5.

In genetically engineered MFS mice with abnormal fibrillin-1, blocking TGF-β, either by TGF-β neutralizing antibody or by the AT_1_ receptor blocker losartan, has been shown to prevent aortic root dilatation, elastic fiber degeneration, and pSmad2 activation [6]. Since dysregulation of TGF-β signaling and activation of the RAS were also observed in fibulin-4 deficient mice, we next investigated the potential therapeutic effect of losartan. To prevent Fibulin-4^R/R^ mice for premature drop-out due to aortic rupture, mice were treated as early as possible. Thus, mice were prenatally treated with placebo, beta-adrenergic receptor blocker propranolol, or AT_1_ receptor blocker losartan. Propranolol, used as standard therapy to slow progression rate of aortic root growth in patients with MFS, served as control agent in an equihypotensive dosage [6]. Cross-sections of ascending aortas collected from Fibulin-4^+/+^ newborn mice, revealed the presence of intact elastic layers (Fig 7A). As expected, placebo-treated Fibulin-4^R/R^ mice showed severe fragmentation and an increased aortic wall thickness in this area (Fig. 7A–B). Treatment of Fibulin-4^R/R^ mice with propranolol did not change elastic fiber fragmentation, but slightly lowered vessel wall thickness. Yet, treatment with losartan improved elastic fiber fragmentation and greatly reduced vessel wall thickness.

Since AT_1_ receptor blockade is contraindicated during pregnancy and aortic aneurysms are usually diagnosed in a more advanced state, we performed a postnatal treatment trial with losartan. While only a minority of the Fibulin4^R/R^ untreated mice reach a lifespan of 120–140 days, losartan-treated Fibulin4^R/R^ animals could reach a lifespan of at least 160–180 days (n = 3), after which they were sacrificed for histological analysis. Postnatal treatment of Fibulin-4^R/R^ mice with losartan did not reduce vessel wall thickness, but contrary, led to aortic wall thickening when compared to placebo-treated Fibulin-4^R/R^ mice (Fig. 7D). This might, at least in part, be due to the increase in age of the losartan-treated animals. There were no signs of active remodeling of the aortic wall due to losartan treatment, since no change in elastic fiber architecture (Fig. 7C) or lumen diameter (Fig. 7F) was observed. To address whether losartan was able to reduce TGF-β signaling in these adult animals we performed pSmad2 staining. We found no reduction in pSmad2 positive cells with 99% of all nuclei stained positive for both groups (Fig. 7E). Since losartan reduced systolic blood pressure to approximately 60 mmHg, we therefore attribute the increased lifespan of the losartan-treated animals to the lower blood pressure measured.

## Discussion

Adult fibulin-4 deficient (Fibulin-4^+/R^ and Fibulin-4^R/R^) mice display gene-dose-dependent elastic fiber fragmentation, dropout of SMCs, and deposition of mucopolysaccharide ground substance in the ECM of the aortic media. The structural changes observed in adult fibulin-4 deficient mice reflect the key histological features of cystic medial degeneration in patients with aortic aneurysm or dissection [1], [28], [29]. In patients, medial degeneration is histologically characterized by fragmentation and loss of elastin, loss of SMCs, and formation of areas devoid of elastin that are filled with amorphous ECM. Cystic medial degeneration characterizes the final common pathway for various processes that affect the integrity of the aortic media. These findings support the use of the fibulin-4 deficient murine model for the study of aortic degeneration and aneurysm formation and its pharmacotherapeutical intervention.

The ECM provides the structural and functional platform of the aorta. In normal healthy aorta, elastin and collagen account for 50% of the dry weight and provide the aortic wall with non-linear elasticity properties [30]. One of the critical elements of the ECM are the elastic lamellae. Elastin is incorporated in elastic fibers on a scaffold of microfibrils. The elastic fibers in normal healthy aorta are arranged in concentric elastic lamellae and, together with vascular SMCs, form lamellar units [31]. Deposition of elastin is not uniform in the aorta, with a decrease in the number of elastin lamellar units from the ascending aorta to the abdominal aorta [30]. The circumferentially aligned collagen and elastin fibers in the aortic media provide tensile strength, permitting the aorta to withstand pulsatile flow and blood pressure delivered by the heart and to limit distal shear stress. The loss of elastic fiber integrity in the aortic wall observed in fibulin-4 deficient mice was associated with an increase in aortic pulse pressure, mainly due to a decline in diastolic blood pressure, reflecting diminished aortic resilience and tensile strength. Similar stiffening of the aortic wall with increased pulse pressure has been found in the well-characterized genetically engineered mouse model of MFS with a mutation in the FBN1 gene (*Fbn1* ^C1039G/+^) and in patients with MFS [32] [33]. The rise in aortic pulse pressure in conjunction with aortic dilatation will further increase arterial wall stress over the cardiac cycle and thereby extend elastic fiber fragmentation. In MFS patients it has been shown that elevated aortic pulse-wave velocity, as a measure for reduced aortic elasticity, is a predictor for aortic dilatation and dissection [34].

The changes in aortic media structure were accompanied by impaired contractile function. Both adrenergic-receptor and receptor-independent vascular contractility were reduced in fibulin-4 deficient aortic rings. The decreased contractile capacity could, at least in part, be explained by the loss of SMCs in fibulin-4 deficient aortas. In addition, loss of fibulin-4 is assumed to disrupt the interaction between elastic fibers and SMCs, leading to alterations in actin cytoskeleton organization [35]. Third, altered calcium signaling may contribute to disturbed vascular contractile capacity. Using aortic transcriptome analysis, we identified altered expression pattern of genes encoding for proteins involved in calcium signaling in Fibulin-4^+/R^ as compared with Fibulin-4^+/+^ aortas. These data indicate that fibulin-4 deletion not only affects aortic media structure, but also affects contractile function, as was also predicted based on fibulin-4 conditional knockout mice [35]. It has been suggested that aortic contractility contributes to the overall tensile strength and structural integrity of the aortic wall [36]. The observed disturbances in the biomechanical properties of the aorta are in line with findings in the genetic mouse model of MFS [37]. The altered load-bearing capacity of the aorta due to disturbances in the synthesis and breakdown of the aortic medial ECM as well as impaired aortic contractility culminates in increased aortic wall stress, which may contribute to dissection and aneurysm formation.

As in the MFS mouse model, the alterations in aortic structure and function were associated with increased TGF-β signaling in adult aneurysmal fibulin-4 deficient mice, as evidenced by a graded increase in the expression of pSmad2, an intracellular mediator of the TGF-β signal, in the aortic media of Fibulin-4^+/R^ and Fibulin-4^R/R^ mice. Augmented TGF-β activation is associated with upregulation of matrix metalloproteinases and degradation of the aortic media, as shown in both MFS mice and in newborn fibulin-4 deficient mice [37], [38], [39]. Furthermore, altered TGF-β signaling has also been reported in humans with cardiovascular malformations due to fibulin-4 deficiency [40]. The importance of TGF-β signaling in aneurysm formation is further supported by the recent demonstration of increased circulating TGF-β concentrations in patients and mice with MFS, and the correlation between increased serum TGF-β and aortic root dilatation [41].

It is still unclear how fibulin-4 deficiency correlates with increased TGF-β signaling. Increased TGF-β production may be due to Ang II [42], [43], [44], [45]. For example, in human vascular SMCs, stimulation with Ang II induced a 6-fold increase in TGF-β production [43]. The contribution of the RAS in the fibulin-4 mouse model was investigated by measuring circulating and renal tissue Ang II. Changes in renal Ang II content mirror changes in the Ang II content of other tissues, including the aorta [17], [27]. However, renal Ang II levels are generally much higher than Ang II levels in blood vessel walls, and can thus be measured with much greater accuracy. Therefore, we determined renal tissue Ang II levels as a reflection of changes in aortic Ang II content in adult fibulin-4 deficient mice. While Ang II levels were preserved in plasma, renal Ang II levels increased with decreasing expression of fibulin-4. Parallel increases in renal AT_1b_ receptor content were observed, although these increases were not yet significant at n = 3–10. Since tissue Ang II levels are determined largely, if not completely, by AT_1a_ and/or AT_1b_ receptor binding and subsequent internalization of extracellularly generated Ang II [17], [27], these data suggest that the increased renal Ang II levels are due to increased renal AT_1_ receptor binding. Importantly, qPCR supported an aortic AT receptor upregulation profile in these mice that was identical to the profile in the kidney, i.e., selective AT_1b_ upregulation. Thus, based on these data it seems reasonable to assume that the vascular Ang II levels, like the renal Ang II levels, are increased in fibulin-4 deficient mice due to increased AT_1_ receptor binding. Alternatively, upregulated tissue Ang II levels may be due to increased renin uptake at tissue sites [46], and thus future studies should investigate vascular (pro)renin receptor density. Evidence is accumulating that the RAS plays an important role in the pathogenesis of aneurysm formation [6], [25], [47], [48], [49]. Ang II and AT_2_ receptor expression are increased in MFS aortic tissue and have been associated with cystic medial degeneration [25]. The increased tissue Ang II levels observed in fibulin-4 deficient mice are in line with these findings and support the role for the RAS in this model.

Drugs that interfere with the RAS may reduce aortic media degeneration. In cultured aortic cells from MFS, angiotensin-converting enzyme inhibition and AT_1_ receptor antagonism significantly inhibited SMC apoptosis [25]. Interestingly, blockade of the AT_1_ receptor by losartan has been shown to diminish TGF-β signaling, with a reduction in free TGF-β levels, tissue expression of TGF-β–responsive genes, and levels of mediators within the TGF-β signaling cascade, and to prevent aortic aneurysm development in the MFS mouse model [6]. Furthermore, treatment with losartan reduced circulating TGF-β levels and slowed the rate of aortic root dilatation both in MFS mice and in MFS patients [13], [41]. Based on these findings, we investigated whether aortic media degeneration in the fibulin-4 aneurysm model is associated with increased TGF-β signaling and could be prevented by the TGF-β antagonist losartan. In addition to interfering with TGF-β signaling, AT_1_ receptor blockade will indirectly stimulate the AT_2_ receptor. Through a negative feedback-mechanism, Ang II levels rise and bind the AT_2_ receptor, which can have positive effects on the vascular remodeling. Results obtained in MFS mice further demonstrate that losartan is able to improve phenylephrine-induced contractility [50].

AT_1_ receptor blocker losartan, but not by the β-blocker propranolol, prevented elastic fiber fragmentation and disarray in the aortic media of newborn Fibulin-4^R/R^ mice. Treatment of established aortic aneurysms in adult fibulin-4 mice with these losartan doses (0.6 g/L) did not affect elastic fiber architecture. Thus, losartan was only able to prevent aortic wall degeneration in newborn Fibulin-4^R/R^ mice. This is opposite to findings in MFS mice, where postnatal treatment did improve aortic wall degeneration [6]. Clearly, there are differences between the mouse models. All Fibulin-4^R/R^ mice suffer from severe aortic aneurysms from birth that are prone to rupture, resulting in a tremendous reduction in lifespan as compared to their wild type littermates. MFS mice start to develop aortic aneurysms at the age of two months with variable severity of the aneurysm, and have a normal lifespan [51]. We hypothesize that aortic damage of Fibulin-4^R/R^ mice at the age of 5.5 weeks is too severe to regress or prevent further aortic growth with losartan treatment. Therefore, no difference in vessel lumen could be observed between placebo- or losartan-treated Fibulin-4^R/R^ mice. Most importantly, lifespan of adult Fibulin-4^R/R^ mice treated with losartan largely improved, accompanied with an increase with vessel wall thickness. Thus, thickening of the aortic wall might prevent aortic rupture. Postnatal losartan treatment of Fibulin-4R/R animals neither resulted in improved vessel wall structure nor in reduced TGF-β signaling, arguing against an active remodeling of the aorta.. Thus, the improved lifespan seem to be a result of a reduced haemodynamic stress, evidenced by a lower blood pressure. Whether results are specific for AT1 receptor blockers and/or inhibitors of the renin-angiotensin system or whether similar effects can be obtained with other blood pressure lowering agents has to be evaluated.

The present study is the first to show that losartan is effective in the prevention of non-MFS based aortic aneurysms. For established aortic aneurysms, losartan proved to largely improve lifespan, accompanied with a (preventive) thickening of the aortic wall. Together with previous reports, these data suggest that the antihypertensive drug losartan, an AT_1_ receptor blocker that blunts TGF-β activation, may be an effective drug in the early secondary prevention of aortic media degeneration and aneurysm formation.

## Supporting Information

Table S1 **Top ten Ingenuity Canonical Pathways following ANOVA (Fibulin-4^+/R^ vs. Fibulin-4^+/+^).** Top canonical pathways of aortic transcriptome changes in Fibulin-4^+/R^ mice compared to Fibulin-4^+/+^ littermates. When comparing Fibulin-4^+/R^ to Fibulin-4^+/+^ aortas, mostly genes involved in calcium signaling were identified (*).
(DOC)

Table S2 **Top ten Ingenuity Canonical Pathways following Statistical Analysis of Microarrays (Fibulin-4^+/R^ vs. Fibulin-4^+/+^).** Top canonical pathways of aortic transcriptome changes in Fibulin-4^+/R^ mice compared to Fibulin-4^+/+^ littermates. Molecules involved in muscle contractility showed high significance (*). Furthermore, many genes involved in immune responses and infectious diseases were identified.
(DOC)

Table S3 **Top ten Ingenuity Canonical Pathways following ANOVA (Fibulin-4^R/R^ vs. Fibulin-4^+/+^).** Top canonical pathways of aortic transcriptome changes in Fibulin-4^R/R^ mice compared to Fibulin-4^+/+^ littermates. Analysis of Fibulin-4^R/R^ mice revealed mainly genes associated with immunological or infectious diseases. TGF-*β* showed upregulation (*).
(DOC)

Table S4 **Top ten Ingenuity Canonical Pathways following Statistical Analysis of Microarrays (Fibulin-4^R/R^ vs. Fibulin-4^+/+^).** Top canonical pathways of aortic transcriptome changes in Fibulin-4^R/R^ mice compared to Fibulin-4^+/+^ littermates. Mainly genes involved in immune responses and infectious diseases were identified. TGF-*β* showed upregulation (*).
(DOC)
