# Supplementary material for: Impaired Vascular Contractility and Aortic Wall Degeneration in Fibulin-4 Deficient Mice: Effect of Angiotensin II Type 1 (AT1) Receptor Blockade
Source: PLoS One. 2011 Aug 9;6(8):e23411. doi: 10.1371/journal.pone.0023411 (PMC3153486; doi:10.1371/journal.pone.0023411)
Supplement: Table S1 — Top ten Ingenuity Canonical Pathways following ANOVA (Fibulin-4+/R vs. Fibulin-4+/+). Top canonical pathways of aortic transcriptome changes in Fibulin-4+/R mice compared to Fibulin-4+/+ littermates. When comparing Fibulin-4+/R to Fibulin-4+/+ aortas, mostly genes involved in calcium signaling were identified (*). (DOC) [file pone.0023411.s001.doc]

| **Ingenuity Canonical Pathways** | ***p*-value** | **Ratio** | **Genes** |
| --- | --- | --- | --- |
| Calcium Signaling | 3.5*10^-10^ | 0.034 | ACTA1↑*, ATP2A1↑*, MYH1↑*, MYH2↑*, TNNC2↑*, TNNT3↑*, TNNI2↑* |
| Actin Cytoskeleton Signaling | 2.5*10^-6^ | 0.021 | ACTA1↑*, MYH1↑*, MYH2↑*, MYLPF↑, PIK3CD↑ |
| C21-Steroid Hormone Metabolism | 2.0*10^-4^ | 0.029 | HSD11B1↑, HSD3B2↑ |
| Tight Junction Signaling | 5.5*10^-4^ | 0.018 | ACTA1↑*, MYH1↑*, MYH2↑* |
| Leukocyte Extravasation Signaling | 7.9*10^-4^ | 0.015 | ACTA1↑, NCF2↑, PIK3CD↑ |
| Calcium-induced T Lymphocyte Apoptosis | 1.3*10^-3^ | 0.032 | HLA-DMA↑, ATP2A1↑* |
| Androgen and Estrogen Metabolism | 1.6*10^-3^ | 0.015 | HSD11B1↑, HSD3B2↑ |
| IL-4 Signaling | 2.0*10^-3^ | 0.028 | HLA-DMA↑, PIK3CD↑ |
| Nitric Oxide Signaling in the Cardiovascular System | 2.3*10^-3^ | 0.022 | ATP2A1↑*, PIK3CD↑ |
| CTLA4 Signaling in Cytotoxic T Lymphocytes | 3.0*10^-3^ | 0.023 | HLA-DMA↑, PIK3CD↑ |
