# Supplementary material for: Impaired Vascular Contractility and Aortic Wall Degeneration in Fibulin-4 Deficient Mice: Effect of Angiotensin II Type 1 (AT1) Receptor Blockade
Source: PLoS One. 2011 Aug 9;6(8):e23411. doi: 10.1371/journal.pone.0023411 (PMC3153486; doi:10.1371/journal.pone.0023411)
Supplement: Table S2 — Top ten Ingenuity Canonical Pathways following Statistical Analysis of Microarrays (Fibulin-4+/R vs. Fibulin-4+/+). Top canonical pathways of aortic transcriptome changes in Fibulin-4+/R mice compared to Fibulin-4+/+ littermates. Molecules involved in muscle contractility showed high significance (*). Furthermore, many genes involved in immune responses and infectious diseases were identified. (DOC) [file pone.0023411.s002.doc]

| **Ingenuity Canonical Pathways** | ***p*-value** | **Ratio** | **Genes** |
| --- | --- | --- | --- |
| Antigen Presentation Pathway | 6.3*10^-22^ | 0.359 | B2M↑, CD74↑, HLA-B↑, HLA-C↑, HLA-DMA↑, HLA-DMB↑, HLA-DOA↑, HLA-DQA1↑, HLA-DQB2↑, HLA-DRB1↑, HLA-E↑, PSMB8↑, PSMB9↑, TAP1↑ |
| Dendritic Cell Maturation | 7.9*10^-17^ | 0.115 | B2M↑, CD86↑, FCGR2A↑, FCGR2B↑, FCGR3A↑,HLA-B↑, HLA-C↑, HLA-DMA↑, HLA-DMB↑, HLA-DOA↑, HLA-DQA1↑, HLA-DQB2↑, HLA-DRB1↑, NFKBIE↑, PIK3CA↓, PIK3CD↑, STAT1↑, STAT4↑, TYROBP↑ |
| Calcium Signaling | 1.6*10^-13^ | 0.087 | ACTA1↑*, ACTC1↓*, ATP2A1↑*, MYH1↑*, MYH2↑*, MYH6↓*, MYH8↑*, MYL1↑*, MYL4↓*, MYL7↓*, RYR1↑*, TNNC1↓*, TNNC2↑*, TNNI2↑*, TNNI3↓*, TNNT2↓*, TNNT3↑*, TRDN↑* |
| CD28 Signaling in T Helper Cells | 6.3*10^-11^ | 0.105 | CARD11↑, CD247↑, CD4↑, CD86↑, HLA-DMA↑, HLA-DMB↑, HLA-DOA↑, NFKBIE↑, PIK3CA↓, PIK3CD↑, PTPRC↑, SYK↑, VAV1↑ |
| Role of NFAT in Regulation of the Immune Response | 1.0*10^-10^ | 0.080 | BLNK↑, CD247↑, CD4↑, CD86↑, FCER1G↑, FCGR2A↑, FCGR2B↑, FCGR3A↑, HLA-DMA↑, HLA-DMB↑, HLA-DOA↑, NFKBIE↑, PIK3CA↓, PIK3CD↑, SYK↑ |
| IL-4 Signaling | 7.8*10^-10^ | 0.139 | B2M↑, HLA-DMA↑, HLA-DMB↑, HLA-DOA↑, HLA-DQA1↑, HLA-DQB2↑, HLA-DRB1↑, INPP5D↑, PIK3CA↓,PIK3CD↑ |
| Natural Killer Cell Signaling | 2.0*10^-9^ | 0.096 | CD247↑, FCER1G↑, FCGR3A↑, INPP5D↑, KLRD1↑, PIK3CA↓, PIK3CD↑, PRKCB↑, SYK↑, TYROBP↑, VAV1↑ |
| B Cell Receptor Signaling | 3.2*10^-9^ | 0.084 | BLNK↑, CD22↑, FCGR2A↑, FCGR2B↑, INPP5D↑, NFKBIE↑, PIK3AP1↑, PIK3CA↓, PIK3CD↑, PRKCB↑, PTPRC↑, SYK↑, VAV1↑ |
| Fcγ Receptor-mediated Phagocytosis in Macrophages and Monocytes | 3.7*10^-9^ | 0.106 | ACTA1↑*, ACTC1↓*, FCGR2A↑, FCGR3A↑, FGR↑, HCK↑, INPP5D↑, PLD4↑, PRKCB↑, SYK↑, VAV1↑ |
| CTLA4 Signaling in Cytotoxic T Lymphocytes | 6.5*10^-9^ | 0.112 | CD247↑, CD4↑, CD86↑, HLA-DMA↑, HLA-DMB↑, HLA-DOA↑, PIK3CA↓, PIK3CD↑, PTPN22↑, SYK↑ |
