# Supplementary material for: Impaired Vascular Contractility and Aortic Wall Degeneration in Fibulin-4 Deficient Mice: Effect of Angiotensin II Type 1 (AT1) Receptor Blockade
Source: PLoS One. 2011 Aug 9;6(8):e23411. doi: 10.1371/journal.pone.0023411 (PMC3153486; doi:10.1371/journal.pone.0023411)
Supplement: Table S3 — Top ten Ingenuity Canonical Pathways following ANOVA (Fibulin-4R/R vs. Fibulin-4+/+). Top canonical pathways of aortic transcriptome changes in Fibulin-4R/R mice compared to Fibulin-4+/+ littermates. Analysis of Fibulin-4R/R mice revealed mainly genes associated with immunological or infectious diseases. TGF-β showed upregulation (*). (DOC) [file pone.0023411.s003.doc]

| **Ingenuity Canonical Pathways** | ***p*-value** | **Ratio** | **Genes** |
| --- | --- | --- | --- |
| Complement System | 3.5*10^-7^ | 0.167 | C1QA↑, C1QB↑, C1QC↑, C3AR1↑, C5AR1↑, CFB↑ |
| Role of Pattern Recognition Receptors in Recognition of Bacteria and Viruses | 9.3*10^-7^ | 0.091 | C1QA↑, C1QB↑, C1QC↑, C3AR1↑, C5AR1↑, CASP1↑, IRF1↑, TLR2↑ |
| IL-12 Signaling and Production in Macrophages | 6.2*10^-6^ | 0.061 | ALOX12↑, IRF1↑, MAF↑, PRKCD↑, SPI1↑, STAT1↑, TGFB1↑*, TLR2↑ |
| TREM1 Signaling | 1.5*10^-5^ | 0.087 | CASP1↑, CCL2↑, CD86↑, FCGR2B↑, TLR2↑, TYROBP↑ |
| IL-10 Signaling | 3.2*10^-5^ | 0.085 | CCR1↑, CCR5↑, CD14↑, FCGR2A↑, FCGR2B↑, SOCS3↑ |
| Dendritic Cell Maturation | 4.6*10^-5^ | 0.049 | CD86↑, FCGR2A↑, FCGR2B↑, HLA-B↑, HLA-DMA↑, STAT1↑, TLR2↑, TYROBP↑ |
| Fcγ Receptor-mediated Phagocytosis in Macrophages and Monocytes | 3.5*10^-4^ | 0.058 | ACTG2↓, FCGR2A↑, FGR↑, PLD4↑, PRKCD↑, VAV1↑ |
| Hepatic Fibrosis/ Hepatic Stellate Cell Activation | 1.6*10^-3^ | 0.044 | CCL2↑, CCR5↑, CD14↑, TGFB1↑*, MYH9↑, STAT1↑ |
| Leukocyte Extravasation Signaling | 1.7*10^-3^ | 0.036 | ACTG2↓, CLDN3↑, CYBB↑, MMP14↑, NCF2↑, PRKCD↑, VAV1↑ |
| Natural Killer Cell Signaling | 1.9*10^-3^ | 0.044 | CD300A↑, FCER1G↑, PRKCD↑, TYROBP↑, VAV1↑ |
