# Supplementary material for: Impaired Vascular Contractility and Aortic Wall Degeneration in Fibulin-4 Deficient Mice: Effect of Angiotensin II Type 1 (AT1) Receptor Blockade
Source: PLoS One. 2011 Aug 9;6(8):e23411. doi: 10.1371/journal.pone.0023411 (PMC3153486; doi:10.1371/journal.pone.0023411)
Supplement: Table S4 — Top ten Ingenuity Canonical Pathways following Statistical Analysis of Microarrays (Fibulin-4R/R vs. Fibulin-4+/+). Top canonical pathways of aortic transcriptome changes in Fibulin-4R/R mice compared to Fibulin-4+/+ littermates. Mainly genes involved in immune responses and infectious diseases were identified. TGF-β showed upregulation (*). (DOC) [file pone.0023411.s004.doc]

| **Ingenuity Canonical Pathways** | ***p*-value** | **Ratio** | **Genes** |
| --- | --- | --- | --- |
| Role of Pattern Recognition Receptors in Recognition of Bacteria and Viruses | 7.9*10^-12^ | 0.140 | C1QA↑, C1QB↑, C1QC↑, C5AR1↑, C3AR1↑, CASP1↑, CLEC7A↑, IRF1↑, OAS1↑, SYK↑, TLR1↑, TLR2↑ |
| Complement System | 1.0*10^-8^ | 0.194 | C1QA↑, C1QB↑, C1QC↑, C3AR1↑, C4B↑, C5AR1↑, CFB↑ |
| Atherosclerosis Signaling | 4.3*10^-8^ | 0.091 | ALOX12↑, ALOX15↑, CCL2↑, CCR2↑, ITGB2↑, MSR1↑, SELP↑, TGFB1↑*, VCAM1↑, WISP2↑ |
| Dendritic Cell Maturation | 7.6*10^-8^ | 0.063 | FCER1G↑, FCGR1A↑, FCGR2A↑, FCGR2B↑, FCGR3A↑, TLR2↑, HLA-B↑, HLA-C↑, HLA-DMA↑,TNFRSF1B↑, TYROBP↑ |
| Fcy Receptor-mediated Phagocytosis in Macrophages and Monocytes | 2.9*10^-7^ | 0.089 | HCK↑, FCGR1A↑, FCGR2A↑, FCGR3A↑, FGR↑, PLD4↑, PRKCD↑, SYK↑, VAV1↑ |
| Role of Macrophages, Fibroblasts and Endothelial Cells in Rheumatoid Arthritis | 4.2*10^-7^ | 0.044 | C5AR1↑, CCL2↑, CFB↑, FCGR1A↑, FCGR3A↑, FOS↑, FRZB↑, LRP1↑, PRKCD↑, SOCS3↑, TGFB1↑*, TLR1↑, TLR2↑, TNFRSF1B↑, VCAM1↑ |
| IL-12 Signaling and Production in Macrophages | 5.6*10^-7^ | 0.067 | ALOX12↑, ALOX15↑, FOS↑, IRF1↑, MAF↑, PRKCD↑, SPI1↑, TGFB1↑*, TLR2↑ |
| Systemic Lupus Erythematosus Signaling | 8.7*10^-7^ | 0.060 | CD72↑, FCER1G↑, FCGR1A↑, FCGR2A↑, FCGR2B↑,FCGR3A↑, FOS↑, HLA-B↑,HLA-C↑ |
| TREM1 Signaling | 9.5*10^-7^ | 0.101 | CASP1↑, CCL2↑, FCGR2B↑, LAT2↑, TLR1↑, TLR2↑,TYROBP↑ |
| IL-10 Signaling | 2.0*10^-6^ | 0.100 | CCR1↑, CCR5↑,CD14↑, FCGR2A↑,FCGR2B↑, FOS↑, SOCS3↑ |
